# Target Localization with Unknown Transmit Power and Path-Loss Exponent Using a Kalman Filter in WSNs

**DOI:** 10.3390/s20226582

**Published:** 2020-11-18

**Authors:** SeYoung Kang, TaeHyun Kim, WonZoo Chung

**Affiliations:** 1 Division of Computer and Communications Engineering, Korea University, Seoul 02841, Korea; sykang0229@korea.ac.kr; 2 Agency for Defense Development, Daejeon 34186, Korea; thkimc@hanmail.net

**Keywords:** wireless sensor networks (WSNs), target localization, received signal strength (RSS), angle of arrival (AOA), transmit power (TP), path-loss exponent (PLE), Kalman filter (KF)

## Abstract

We present a novel hybrid localization algorithm for wireless sensor networks in the absence of knowledge regarding the transmit power and path-loss exponent. Transmit power and the path-loss exponent are critical parameters for target localization algorithms in wireless sensor networks, which help extract target position information from the received signal strength. In the absence of information on transmit power and path-loss exponent, it is critical to estimate them for reliable deployment of conventional target localization algorithms. In this paper, we propose a simultaneous estimation of transmit power and path-loss exponent based on Kalman filter. The unknown transmit power and path-loss exponent are estimated using a Kalman filter with the tentatively estimated target position based solely on angle information. Subsequently, the target position is refined using a hybrid method incorporating received signal strength measurements based on the estimated transmit power and path-loss exponent. Our proposed algorithm accurately estimates transmit power and path-loss exponent and yields almost the same target position accuracy as the simulation results confirm, as the hybrid target localization algorithms with known transmit power and path-loss exponent. Simulation results confirm the proposed algorithm achieves 99.7% accuracy of the target localization performance with known transmit power and path-loss exponent, even in the presence of severe received signal strength measurement noise.

## 1. Introduction

Wireless sensor networks (WSNs) are networks comprising spatially spread sensor (or anchor) nodes equipped with sensors for detecting physical environments [1,2]. Recently, considerable attention has been devoted toward the localization of a target of interest based on the measurements of sensors in WSNs [1,2,3,4,5,6,7,8,9,10]. In WSNs, target localization involves estimating the position of a target based on sensor measurements, i.e., received signal strength (RSS) and the angle of arrival (AOA); it plays a crucial role in diverse location-awareness services [10,11]. RSS-based localization algorithms have been reported in [12,13,14], while AOA-based localization algorithms were investigated in [15,16,17]. However, as WSNs were originally not used for location-awareness services, the challenge lies in dealing with the measurement errors in RSS and AOA measurements. To improve the accuracy of target position estimation, hybrid localization algorithms combining both RSS and AOA measurements have been recently investigated in [18,19,20,21,22,23,24].

A majority of these localization algorithms, except for those based solely on AOA measurements, assume that the distance between an anchor and the target can be determined precisely using the measured RSS, which is tantamount to assuming perfect knowledge regarding the transmit power (TP) and path-loss exponent (PLE). However, in practice, prior knowledge of the TP and PLE is not always feasible. In the absence of exact information on TP or PLE, RSS measurements cannot be used reliably for target localization, as they can result in significant performance degradation of RSS-based algorithms. Hence, several studies have focused on RSS-based target localization algorithms for unknown TP or PLE [25,26,27,28,29].

Simultaneous estimation of target position and unknown TP from RSS data was proposed in [25] using semidefinite programming (SDP). Two-step closed-form solutions for the target and unknown TP from RSS data was proposed in [26].

On the contrary, several iterative approaches for estimating target position and TP or PLE for RSS-based algorithms have been proposed with various optimization criteria ([27,28,29]). In [27], the target position is estimated using weighted least squares (WLS) with an initial guess of unknown TP or PLE; subsequently, both target position and TP or PLE are iteratively updated. In [28], SDP optimization techniques were used for an iterative update of the target position and TP and PLE estimates from a random initial guess. In [29], the WLS algorithm and the Levenberg–Marquardt (LM) method were used for an iterative update of target positions under unknown TP and PLE.

Hybrid target localization algorithms for unknown TP or PLE have also been developed [30,31,32,33]. The target position and unknown TP were iteratively estimated in the maximum likelihood formulation using the second-order cone programming (SOCP) algorithm [30]. Hybrid localization with unknown PLE was investigated in [31]. By relating the target position and PLE through a least squares (LS) relation, PLE was estimated via a generalized pattern search, and the target position was estimated via WLS using the estimated PLE. In [32], Generalization of the trust region subproblem (GTRS) optimization technique was applied to obtain initial estimates of the target position and TP via WLS. Subsequently, TP was re-computed from the estimated target position, and the target position was finally refined by reusing the WLS. In [33], the simultaneous estimation of target position and unknown TP as a one-shot WLS optimization has been proposed.

These existing hybrid target localization algorithms, in the absence of information on TP or PLE, rely on exhaustive maximum likelihood estimator (MLE) approximation or iterative updates of the target position and TP or PLE; however, this approach requires significant computational resources. Furthermore, as RSS measurements generally suffer from severe noise, the overall performances of existing hybrid target localization algorithms with unknown TP or PLE are inferior to that of the cutting-edge AOA-only target localization algorithm. In practice, it is difficult to precisely determine both TP and PLE; however, all existing hybrid algorithms dealing with imperfect knowledge regarding TP or PLE assume that either TP or PLE is unknown. Therefore, the development of a hybrid target localization algorithm that does not depend on prior knowledge of both TP and PLE is essential.

In this paper, we propose a novel hybrid target localization scheme for unknown TP and PLE. In the proposed approach, TP and PLE are simultaneously estimated and updated using a Kalman filter (KF) based on the tentatively estimated target position based on AOA-only measurements. The Kalman filter is famous for its robust estimation performance under severe dynamic noise environments [34,35].

Subsequently, RSS measurements are incorporated to obtain the refined target position using the error covariance weighted least squares (EC-WLS) algorithm [24]. Unlike existing iterative methods for unknown TP or PLE, since the proposed algorithm using KF efficiently utilizes a time average of RSS measurements, the proposed algorithm significantly reduces the noise in RSS measurements. Consequently, the proposed algorithm yields accurate TP and PLE estimations and almost the same target position accuracy as the hybrid algorithms with known TP and PLE, even in the presence of severe RSS measurement noise. In this paper, the main contribution is the development of hybrid RSS/AOA localization with unknown TP and PLE that is similar in performance of hybrid RSS/AOA localization with known TP and PLE.

The remainder of the paper follows. Section 2 presents the system model and problem formation for target localization in WSNs, and reviews existing hybrid algorithms for unknown TP or PLE. Section 3 presents the proposed algorithm and demonstrates the role of Kalman filter in TP/PLE estimation with an example. Section 4 presents the computational complexity analysis in comparison with existing algorithms. Section 5 presents the performance evaluation of the proposed method, conducted via simulation, as compared with existing methods. Section 6 describes practical considerations and limitations of the proposed algorithm with performance evaluation under multipath and multiple interference. Finally, Section 7 presents the conclusions of this study.

## 2. System Model and Related Work

In this section, the system model and problem formation for target localization based on RSS and AOA in wireless sensor networks are described. A general linear target estimation approaches with known TP and PLE are introduced and exiting related studies on target localization schemes for unknown TP or PLE have been formally described.

### 2.1. System Model

**Figure 1:**
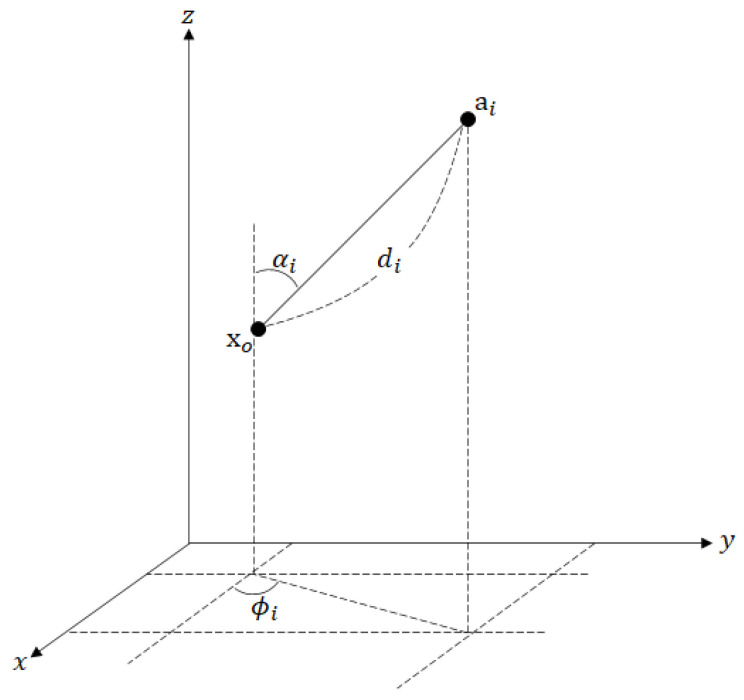
The relation between anchor and target in a 3-D space.

This section introduces the mathematical formulation of the hybrid target localization scheme utilizing both RSS and AOA measurements in WSNs. Let $a_{i}={\left[a_{ix},a_{iy},a_{iz}\right]}^{T}\in R^{3}$ be the known position of the *i*-th sensor (anchor) node for i=1,⋯,N and $x_{o}={\left[x_{ox},x_{oy},x_{oz}\right]}^{T}$ be the unknown location of the sensor (target). The target emits a signal; each node receives the signal from the target along the line-of-sight (LoS) path and measures the RSS, azimuth angle, and elevation angle to the target, which are denoted by ${\hat{P}}_{i}$, ${\hat{\phi }}_{i}$, and ${\hat{\alpha }}_{i}$, respectively (Figure 1).

These RSS and AOA information are sent to a central node (processor) where the target position is estimated. We assume the following assumptions for the target localization in WSNs based on RSS and AOA measurements:

A1. There is only a single sensor node with unknown position (target).
A2. All anchor nodes are equipped with a device that can measure the AOA (directional antenna or antenna arrays) and RSS.
A3. All anchor nodes are fixed at known positions and can transfer RSS/AOA measurements to a central node (processor).
A4. All measurement errors are i.i.d. zero-mean Gaussian noise with unknown variances which are the same for all anchor nodes.
A5. All nodes are in line-of-sight with respect to the target.
A6. All nodes are located at a homogeneous environment so that the path-loss exponent (PLE) is the same for all nodes.

The received power is related to distance by a path-loss model; in the absence of noise, the RSS, $P_{i}$, at the distance, $d_{i}$, from the target is modeled as [36],

(1) $$\begin{array}{c} P_{i}=P_{T}{\left(\frac{d_{0}}{d_{i}}\right)}^{\gamma }10^{-\frac{L_{0}}{10}}, \end{array}$$

where $P_{T}$ is the transmit power of the target, γ denotes the PLE, and $L_{0}$ is the power loss at a reference distance $d_{0}$. Let us define $P_{0}:=P_{T}-L_{0}$, and refer to it as the TP, instead of $P_{T}$. On setting $d_{0}$ as the unit length, the following noisy model in the dB scale is obtained:

(2) $$\begin{array}{c} {\hat{P}}_{i}=P_{0}-10\gamma \log_{10}d_{i}+n_{i}, \end{array}$$

where $n_{i}$ is a zero-mean Gaussian noise with variance, $\sigma_{n_{i}}^{2}$, i.e., $n_{i}$∼$N(0,\sigma_{n_{i}}^{2})$. In (2), the RSS, ${\hat{P}}_{i}$, is a function of the distance, $d_{i}$, assuming the TP, $P_{0}$, and the PLE, γ, are known. The distance estimate, ${\hat{d}}_{i}$, from the RSS, ${\hat{P}}_{i}$, is expressed as

(3) $$\begin{array}{c} {\hat{d}}_{i}=10^{\frac{P_{0}-{\hat{P}}_{i}}{10\gamma }}. \end{array}$$

In the presence of noise, the azimuth angle, ${\hat{\phi }}_{i}$, and the elevation angle, ${\hat{\alpha }}_{i}$, from the target are, respectively, modeled as

(4) $$\begin{array}{c} \begin{array}{cc} {\hat{\phi }}_{i} & =\tan^{-1}\left(\frac{x_{oy}-a_{iy}}{x_{ox}-a_{ix}}\right)+m_{i}\\ {\hat{\alpha }}_{i} & =\cos^{-1}\left(\frac{x_{oz}-a_{iz}}{\left\|x_{o}-a_{i}\right\|}\right)+v_{i} \end{array},\;\;\mathrm{for}\;i=1,\cdots ,N \end{array}$$

where $m_{i}$ and $v_{i}$ are zero-mean Gaussian noise with variance, i.e., $m_{i}$∼$N(0,\sigma_{m_{i}}^{2})$ and $v_{i}$∼$N(0,\sigma_{v_{i}}^{2})$.

When the TP and PLE are known, the MLE for the target position based on RSS/AOA measurements, ${\hat{x}}_{ML}$, is derived as follows [18]:

(5) $$\begin{array}{c} {\hat{x}}_{ML}=\arg\min_{x_{o}}\sum_{i}f_{i}\left({\hat{P}}_{i},{\hat{\phi }}_{i},{\hat{\alpha }}_{i},x_{o}\right) \end{array}$$

where

(6) $$\begin{array}{c} \begin{array}{cc} f_{i}\left({\hat{P}}_{i},{\hat{\phi }}_{i},{\hat{\alpha }}_{i},x_{o}\right) & =\frac{1}{\sigma_{n_{i}}^{2}}{\left({\hat{P}}_{i}-P_{0}+10\gamma \log_{10}\frac{\left\|x_{o}-a_{i}\right\|}{d_{0}}\right)}^{2}\\ & +\frac{1}{\sigma_{m_{i}}^{2}}{\left({\hat{\phi }}_{i}-\tan^{-1}\left(\frac{x_{oy}-a_{iy}}{x_{ox}-a_{ix}}\right)\right)}^{2}\\ & +\frac{1}{\sigma_{v_{i}}^{2}}{\left({\hat{\alpha }}_{i}-\cos^{-1}\left(\frac{x_{oz}-a_{iz}}{\left\|x_{o}-a_{i}\right\|}\right)\right)}^{2} \end{array}\;\;\mathrm{for}\;i=1,\cdots ,N. \end{array}$$

As a closed-form solution of this ML equation is not feasible, several sub-optimal linear estimators have been proposed. All linear estimates are derived from one of the following relations between the target position and the RSS/AOA measurements:

(i) From the relation between Cartesian and spherical coordinates, a direct estimation of the target position (${\hat{x}}_{o}$) can be obtained from the distance estimate (${\hat{d}}_{i}$) and the observed angles (${\hat{\phi }}_{i},{\hat{\alpha }}_{i}$):
  (7) $$\begin{array}{c} \begin{array}{cc} {\hat{x}}_{ox} & =a_{ix}+{\hat{d}}_{i}\cos\left({\hat{\phi }}_{i}\right)\sin\left({\hat{\alpha }}_{i}\right),\\ {\hat{x}}_{oy} & =a_{iy}+{\hat{d}}_{i}\sin\left({\hat{\phi }}_{i}\right)\sin\left({\hat{\alpha }}_{i}\right),\\ {\hat{x}}_{oz} & =a_{iz}+{\hat{d}}_{i}\cos\left({\hat{\alpha }}_{i}\right), \end{array}\;\mathrm{for}\;i=1,\cdots ,N \end{array}$$
  The LS solution of the equation is proposed in [18], and its WLS solution, which is referred to as the WLLS solution, is presented in [31].
(ii) From the maximum likelihood Function (6), the following linear equation can be derived via several non-linear transforms ([33]):
  (8) $$\begin{array}{c} \begin{array}{cc} \lambda_{i}u_{i}^{T}\left(x_{o}-a_{i}\right)-\eta d_{0} & =\varepsilon_{i1}\\ c_{i}^{T}\left(x_{o}-a_{i}\right) & =\varepsilon_{i2}\\ {\left({𝟙}^{T}-u_{i}\cos\left({\hat{\alpha }}_{i}\right)\right)}^{T}\left(x_{o}-a_{i}\right) & =\varepsilon_{i3} \end{array},\;\mathrm{for}\;i=1\cdots N \end{array}$$
  where $\lambda_{i}:=10^{\frac{{\hat{P}}_{i}}{10\gamma }},\;\eta :=10^{\frac{P_{0}}{10\gamma }},\;c_{i}:={\left[-\sin\left({\hat{\phi }}_{i}\right),\cos\left({\hat{\alpha }}_{i}\right),\;0\right]}^{T}$, $𝟙:={\left[0,0,1\right]}^{T}$, $u_{i}:={\left[\cos\left({\hat{\phi }}_{i}\right)\sin\left({\hat{\alpha }}_{i}\right),\sin\left({\hat{\phi }}_{i}\right)\sin\left({\hat{\alpha }}_{i}\right),\cos\left({\hat{\alpha }}_{i}\right)\right]}^{T}$, and $\varepsilon_{ij}$ denotes parameter errors.
  The WLS solution based on range-based weights, referred to as the target-range WLS (TR-WLS) solution, is presented in [33], while the WLS solution based on the approximated error covariance matrix, referred to as the EC-WLS, is presented in [24]. The EC-WLS achieves state-of-the-art target estimation accuracy performance in terms of mean squared error (MSE) [24].

### 2.2. Problem Formation

The existing RSS/AOA localization algorithms estimate the target position when TP and PLE are known. However, as $d_{i}$ in (7) and $\lambda_{i}$ and η in (8) are not available when TP or PLE is unknown, existing hybrid target localization algorithms are subject to failure. Since incorrect TP and PLE degrade the accuracy of a hybrid target estimation algorithm, it is important to accurately estimate TP and PLE first when they are not available.

When the target position is known, TP and PLE can be easily estimated from the received signal power using a linear equation. Given the target position, $x_{o}$, the distance between the target and the *i*-th node, $d_{i}$, is given as

(9) $$\begin{array}{c} d_{i}=\left\|x-a_{i}\right\| \end{array}$$

Using the relation between the distance ($d_{i}$) and the RSS ($P_{i}$) in (2), we obtain a linear equation for the unknown TP and PLE, $z:={\left[P_{0},\gamma \right]}^{T}$, as

(10) $$\begin{array}{c} p=Hz+n \end{array}$$

where

(11) $$\begin{array}{c} p=\left[\begin{array}{c} {\hat{P}}_{1}\\ \vdots \\ {\hat{P}}_{N} \end{array}\right],H=\left[\begin{array}{cc} 1 & -10\log_{10}\left(d_{1}\right)\\ \vdots & \vdots \\ 1 & -10\log_{10}\left(d_{N}\right) \end{array}\right] \end{array}$$

and $n={\left[n_{1},\cdots ,n_{N}\right]}^{T}$ denotes the RSS measurement errors. Straightforwardly, the LS solution for z can be considered:

(12) $$\begin{array}{c} z_{LS}={\left[{\hat{P}}_{0LS},{\hat{\gamma }}_{LS}\right]}^{T}={\left(H^{T}H\right)}^{-1}H^{T}p. \end{array}$$

However, since the goal of the proposed algorithm is to estimate target position, it is not a valid assumption that the target position is known. Furthermore, even if an acceptable estimate of the target position is given, due to the severe RSS noise, the simple LS solution cannot be used for a hybrid target localization algorithm, as shall be shown later. Therefore, there have been several partial solutions for this challenging problem of estimating target position using RSS measurement under unknown TP and PLE, as summarized in the following.

### 2.3. Related Work

Several hybrid target localization algorithms dealing with imperfect knowledge on TP or PLE have been reported [30,31,32,33]. In [30], a multi-target estimation algorithm and a hybrid target localization algorithm for unknown TP are presented. The SOCP approach has been iteratively applied to the following approximation of MLE, including unknown TP, in order to update the target position and TP until satisfactory convergence is achieved.

(13) $$\begin{array}{c} \begin{array}{cc} {\hat{x}}_{o}= & \arg\min_{x_{o},\eta }\sum_{i=1}^{N}{\left(\frac{\lambda_{i}{\left\|x_{o}-a_{i}\right\|}^{2}-\lambda_{i}^{-1}d_{0}^{2}\eta^{2}}{2\left\|x_{o}-a_{i}\right\|}\right)}^{2}+\sum_{i=1}^{N}{\left(c_{i}^{T}\left(x_{o}-a_{i}\right)\right)}^{2}\\ & +\sum_{i=1}^{N}{\left(\frac{𝟙\left(x_{o}-a_{i}\right)-\cos\left({\hat{\alpha }}_{i}\right)\left\|x_{o}-a_{i}\right\|}{\sin\left({\hat{\alpha }}_{i}\right)\left\|x_{o}-a_{i}\right\|}\right)}^{2} \end{array} \end{array}$$

In [31], a hybrid target localization scheme for unknown PLE is proposed. Based on the relation between target position and PLE, a cost function Ψ(γ) is derived:

(14) $$\begin{array}{c} \Psi \left(\gamma \right)=\left[\left[b\left(x\right),b\left(y\right),b\left(z\right)\right]\left(I_{3N}-A{\left(A^{T}A\right)}^{-1}A^{T}\right){\left[b\left(x\right),b\left(y\right),b\left(z\right)\right]}^{T}\right] \end{array}$$

where A=diag[1,1,1] (1 is a column vector *N* ones) and

(15) $$\begin{array}{c} \begin{array}{cc} b(x) & ={\left[a_{1x}+{\hat{d}}_{1}\cos\left({\hat{\phi }}_{1}\right)\sin\left({\hat{\alpha }}_{1}\right),\cdots ,a_{Nx}+{\hat{d}}_{N}\cos\left({\hat{\phi }}_{N}\right)\sin\left({\hat{\alpha }}_{N}\right)\right]}^{T}\\ b(y) & ={\left[a_{1y}+{\hat{d}}_{1}\sin\left({\hat{\phi }}_{1}\right)\sin\left({\hat{\alpha }}_{1}\right),\cdots ,a_{Ny}+{\hat{d}}_{N}\sin\left({\hat{\phi }}_{N}\right)\sin\left({\hat{\alpha }}_{N}\right)\right]}^{T},\\ b(z) & ={\left[a_{1z}+{\hat{d}}_{1}\cos\left({\hat{\alpha }}_{1}\right),\cdots ,a_{Nz}+{\hat{d}}_{N}\cos\left({\hat{\alpha }}_{N}\right)\right]}^{T} \end{array} \end{array}$$

To obtain the optimal PLE (γ) minimizing the cost function, a generalized pattern search algorithm is used, and the target position is subsequently obtained via an WLS equation using the estimated PLE. These iterative approaches have several drawbacks in practical applications, such as exhaustive computational complexity and unreliable accuracy performance, especially in the presence of severe RSS measurement noise.

In [32], a sub-optimal target position estimator based on GTRS, referred to as SR-WLS, for unknown TP was studied. GTRS applied to the MLE formulation yields a WLS optimization based on RSS-based weights for the target position, squared norm of target position, TP, and square of TP; $y={\left[x_{o}^{T},{\left\|x_{o}\right\|}^{2},\eta ,\eta^{2}\right]}^{T}$:

(16) $$\begin{array}{c} \hat{y}=\arg\min_{y}{\left\|W(\tilde{A}y-\tilde{b})\right\|}^{2} \end{array}$$

where $W=I_{3}⊗\mathrm{diag}\left(w\right)$, $w=[w_{i}]$, $w_{i}=1-\frac{P_{i}}{\sum_{j}P_{j}}$ and

(17) $$\begin{array}{c} \tilde{A}=\left[\begin{array}{cccc} -2\lambda_{1}^{2}a_{1}^{T} & \lambda_{1}^{2} & 0 & -d_{0}^{2}\\ \vdots & \vdots & \vdots & \vdots \\ -2\lambda_{N}^{2}a_{N}^{T} & \lambda_{N}^{2} & 0 & -d_{0}^{2}\\ c_{1}^{T} & 0 & 0 & 0\\ \vdots & \vdots & \vdots & \vdots \\ c_{N}^{T} & 0 & 0 & 0\\ \lambda_{1}{𝟙}^{T} & 0 & -d_{0}\cos\left({\hat{\alpha }}_{1}\right) & 0\\ \vdots & \vdots & \vdots & \vdots \\ \lambda_{1}{𝟙}^{T} & 0 & -d_{0}\cos\left({\hat{\alpha }}_{N}\right) & 0 \end{array}\right],\tilde{b}=\left[\begin{array}{c} -\lambda_{1}^{2}{\left\|a_{1}\right\|}^{2}\\ \vdots \\ -\lambda_{N}^{2}{\left\|a_{N}\right\|}^{2}\\ c_{1}^{T}a_{1}\\ \vdots \\ c_{N}^{T}a_{N}\\ \lambda_{1}𝟙a_{1}\\ \vdots \\ \lambda_{N}𝟙a_{N} \end{array}\right] \end{array}$$

To improve the accuracy of the TP estimate, TP is re-calculated from the TP-target position relation using the estimated target position.

(18) $$\begin{array}{c} {\hat{P}}_{0}=\frac{\sum_{i}\left({\hat{P}}_{i}+10\gamma \log_{10}\left\|{\hat{x}}_{o}-a_{i}\right\|\right)}{N} \end{array}$$

Subsequently, the target position is refined using the Equation (17) with the re-calculated TP (${\hat{P}}_{0}$).

In [33], target estimation for unknown TP based on RSS-based WLS optimization treating both target position and TP as parameters to be estimated, i.e., $y={\left[x_{o},\eta \right]}^{T}$, was presented:

(19) $$\begin{array}{c} \hat{y}=\arg\min_{y}{\left\|W(Ay-b)\right\|}^{2} \end{array}$$

where

(20) $$\begin{array}{c} \tilde{A}=\left[\begin{array}{cc} \lambda_{1}u_{1}^{T} & d_{0}\\ \vdots & \vdots \\ \lambda_{N}u_{N}^{T} & d_{0}\\ c_{1}^{T} & 0\\ \vdots & \vdots \\ c_{N}^{T} & 0\\ {\left(\cos\left({\hat{\alpha }}_{1}\right)u_{1}-𝟙\right)}^{T} & 0\\ \vdots & \vdots \\ {\left(\cos\left({\hat{\alpha }}_{N}\right)u_{N}-𝟙\right)}^{T} & 0 \end{array}\right],\tilde{b}=\left[\begin{array}{c} \lambda_{1}u_{1}^{T}a_{1}\\ \vdots \\ \lambda_{N}u_{N}^{T}a_{N}\\ c_{1}^{T}a_{1}\\ \vdots \\ c_{N}^{T}a_{N}\\ {\left(\cos\left({\hat{\alpha }}_{1}\right)u_{1}-𝟙\right)}^{T}a_{1}\\ \vdots \\ {\left(\cos\left({\hat{\alpha }}_{N}\right)u_{N}-𝟙\right)}^{T}a_{N} \end{array}\right] \end{array}$$

This algorithm, referred to as the target-TP WLS (TT-WLS) algorithm, is simple, practical, and achieves the best target position accuracy among the above-mentioned hybrid target localization schemes without TP or PLE. However, even TT-WLS exhibits inferior performance in comparison with the AOA-only target localization algorithm derived from the state-of-art EC-WLS algorithm in the presence of severe RSS measurement, as discussed in the simulation section. Therefore, it is important to develop a hybrid target estimation algorithm with unknown TP or PLE that exceeds the performance of AOA-only target localization algorithms.

## 3. Proposed Method

This section presents the proposed hybrid localization with unknown TP and PLE based on a KF. The proposed algorithm comprises three steps: (i) Compute the initial target position using the EC-WLS based on AOA measurements only; (ii) estimate TP and PLE using the Kalman Filter; (iii) estimate the refined target position using EC-WLS based on RSS/AOA measurements using the estimated TP and PLE. We refer to the proposed algorithm as Kalman filter ECWLS (KF-WLS) algorithm.

### 3.1. Estimation of Initial Target Position Using EC-WLS Based on AOA’s

To estimate the initial target estimation without TP or PLE, only the equations related to AOA measurements are considered from (8):

(21) $$\begin{array}{c} \begin{array}{cc} c_{i}^{T}\left(x_{o}-a_{i}\right) & =\varepsilon_{i2}\\ {\left({𝟙}^{T}-u_{i}\cos\left({\hat{\alpha }}_{i}\right)\right)}^{T}\left(x_{o}-a_{i}\right) & =\varepsilon_{i3} \end{array},\;\mathrm{for}\;i=1\cdots N, \end{array}$$

In a matrix form,

(22) $$\begin{array}{c} Bx_{o}-c=\varepsilon \end{array}$$

where

(23) $$\begin{array}{c} B=\left[\begin{array}{c} c_{1}^{T}\\ \vdots \\ c_{N}^{T}\\ {\left(\cos\left({\hat{\alpha }}_{1}\right)u_{1}-𝟙\right)}^{T}\\ \vdots \\ {\left(\cos\left({\hat{\alpha }}_{N}\right)u_{N}-𝟙\right)}^{T} \end{array}\right],\;c=\left[\begin{array}{c} c_{1}^{T}a_{1}\\ \vdots \\ c_{N}^{T}a_{N}\\ {\left(\cos\left({\hat{\alpha }}_{1}\right)u_{1}-𝟙\right)}^{T}a_{1}\\ \vdots \\ {\left(\cos\left({\hat{\alpha }}_{N}\right)u_{N}-𝟙\right)}^{T}a_{N} \end{array}\right], \end{array}$$

and $\varepsilon ={\left[\begin{array}{cccccc} \varepsilon_{12} & \cdots & \varepsilon_{N2} & \varepsilon_{13} & \cdots & \varepsilon_{N3} \end{array}\right]}^{T}$. We can apply existing WLS approaches for this dimension reduction form; the EC-WLS approach [24], which uses the estimated error covariance for weights and achieves the best estimation accuracy among existing hybrid algorithms, is employed:

(24) $$\begin{array}{c} {\hat{x}}_{AOA}={\left(B^{T}C_{A}^{-1}B\right)}^{-1}B^{T}C_{A}^{-1}c, \end{array}$$

where $C_{A}$ is the approximated error covariance matrix for AOA measurements, which is detailed in Appendix A.

### 3.2. Kalman Filter-Based Estimation of Transmit Power and Path-Loss Exponent

From ${\hat{x}}_{AOA}$ in (24), the estimated distance between the target and the *i*-th node, ${\hat{d}}_{i}$, is expressed as

(25) $$\begin{array}{c} {\hat{d}}_{i}=\left\|{\hat{x}}_{AOA}-a_{i}\right\| \end{array}$$

Let $z={\left[P_{0},\gamma \right]}^{T}$ be the TP and PLE to be estimated. Then, as presented in Section 2.2, we have

(26) $$\begin{array}{c} p=Hz+n \end{array}$$

where

(27) $$\begin{array}{c} p=\left[\begin{array}{c} \hat{\;}P_{1}\\ \vdots \\ {\hat{P}}_{N} \end{array}\right],H=\left[\begin{array}{cc} 1 & -10\log_{10}\left({\hat{d}}_{1}\right)\\ \vdots & \vdots \\ 1 & -10\log_{10}\left({\hat{d}}_{N}\right) \end{array}\right] \end{array}$$

and $n={\left[n_{1},\cdots ,n_{N}\right]}^{T}$ denotes the RSS measurement errors.

To solve the above equation iteratively over time samples, we apply a Kalman Filter. Let ${\hat{P}}_{i}\left[k\right]$, ${\hat{P}}_{0}\left[k\right]$, γ[k], and $n_{i}\left[k\right]$ denote the RSS measured at time *k* on the node *i*, TP and PLE at time *k*, and measurement noise in dB at time k for the node *i*, respectively. Subsequently, the Equation (26) can be generalized to the following state-space model for TP and PLE:

(28) $$\begin{array}{c} \begin{array}{cc} z_{k} & =z_{k-1},\\ p_{k} & =Hz_{k}+n_{k}, \end{array} \end{array}$$

where

(29) $$\begin{array}{c} z_{k}=\left[\begin{array}{c} P_{0}\left[k\right]\\ \gamma [k] \end{array}\right],p_{k}=\left[\begin{array}{c} {\hat{P}}_{1}\left[k\right]\\ \vdots \\ {\hat{P}}_{N}\left[k\right] \end{array}\right],n_{k}=\left[\begin{array}{c} n_{1}\left[k\right]\\ \vdots \\ n_{N}\left[k\right] \end{array}\right] \end{array}$$

In this state-space model, time invariance of the target position is assumed. When the target position is varies with time, *H* can be replaced by $H_{k}$ with the distance of the target position from the *i*-th node at time *k*, i.e., ${\hat{d}}_{i}\left[k\right]$. Based on this state-space model (28), the KF operates in two steps: predict and update [34]. The two steps are specified as follows:

(30) $$\begin{array}{c} \begin{array}{cc} Predict & :\;\left\{\begin{array}{c} z_{k|k-1}=z_{k-1}\\ Q_{k|k-1}=Q_{k-1} \end{array}\right.\\ Update & :\;\left\{\begin{array}{c} z_{k}=z_{k|k-1}+K_{k}\left(p_{k}-Hz_{k|k-1}\right)\\ K_{k}=Q_{k|k-1}H^{T}{\left(HQ_{k|k-1}H^{T}+R\right)}^{-1}\\ Q_{k}=\left(I-K_{k}H\right)Q_{k|k-1} \end{array}\right. \end{array} \end{array}$$

where $R=\mathrm{diag}[{\hat{\sigma }}_{n_{1}}^{2},\cdots ,{\hat{\sigma }}_{n_{N}}^{2}]$, the estimated variance of RSS measurement noises using $x_{AOA}$ and $z_{LS}$,

(31) $$\begin{array}{c} {\hat{\sigma }}_{n_{i}}^{2}=\frac{1}{N}\sum_{i=1}^{N}{\left({\hat{P}}_{i}-{\hat{P_{0}}}_{LS}+10{\hat{\gamma }}_{LS}\log_{10}\left\|x_{AOA}-a_{i}\right\|\right)}^{2}. \end{array}$$

The initial value of $z_{0}$ is set by $z_{LS}$ in (12), and $Q_{0}$ is set by a 2×2 identity matrix $I_{2}$.

### 3.3. Example of KF Based TP and PLE Estimation

In the following example, we demonstrate the performance of Kalman filter by tracing the instantaneous parameter error, ${\hat{\theta }}_{k}$, which is defined as

(32) $$\hat{\theta }\left({\hat{P}}_{0k}\right)={\left|P_{0}-{\hat{P}}_{0k}\right|}^{2},$$

(33) $$\hat{\theta }\left({\hat{\gamma }}_{k}\right)={\left|\gamma -{\hat{\gamma }}_{k}\right|}^{2},$$

where ${\hat{P}}_{0k}$ and ${\hat{\gamma }}_{k}$ are the estimates of the true TP ($P_{0}$) and PLE (γ) at time *k*.

We consider a scenario that a target and six anchor nodes (N=6) are arbitrarily located inside a box with an edge length B=15 m. The true TP and PLE are given as $P_{0}=-10$ dBm and γ=2.5, respectively.

**Figure 2:**
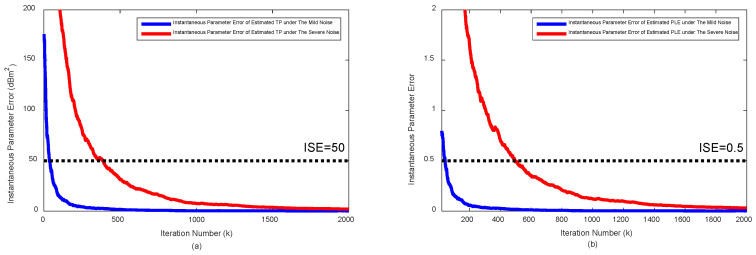
Parameter error vs. iteration under the mild noise ($\sigma_{n_{i}}=2$ dB, $\sigma_{m_{i}}=\sigma_{v_{i}}=10$ deg) and the severe noise ($\sigma_{n_{i}}=6$ dB, $\sigma_{m_{i}}=\sigma_{v_{i}}=10$ deg): (**a**) for TP, $\hat{\theta }\left(P_{0k}\right)$, and (**b**) for path-loss exponent (PLE), $\hat{\theta }\left(\gamma_{k}\right)$.

Figure 2 shows the $\hat{\theta }\left({\hat{P}}_{0k}\right)$ and $\hat{\theta }\left({\hat{\gamma }}_{k}\right)$ over the iteration under mild RSS noise ($\sigma_{n_{i}}=2$ dB, $\sigma_{m_{i}}=\sigma_{v_{i}}=10$ deg) and severe RSS noise ($\sigma_{n_{i}}=6$ dB, and $\sigma_{m_{i}}=\sigma_{v_{i}}=10$ deg), respectively.

The initial TP, ${\hat{P}}_{00}$, and PLE ${\hat{\gamma }}_{0}$, are set by LS solutions in Equation (12), i.e., ${\hat{P}}_{00}={\hat{P}}_{0LS}$, ${\hat{\gamma }}_{0}={\hat{\gamma }}_{LS}$. Under the presence of RSS noise, the LS solutions are deviated from the true values substantially and with the LS estimates the hybrid target localization algorithm cannot outperform the AOA-only target estimation algorithm even under the mild RSS noise case. As the iteration of Kalman filter proceeds, the parameter error is reduced and eventually converged to 0. In this example, when the parameter errors are smaller than the following numerically found threshold $\hat{\theta }\left({\hat{P}}_{0k}\right)<50$ dBm${}^{2}$ and PLE $\hat{\theta }\left(\gamma_{k}\right)<0.5$, the hybrid target localization algorithm with the TP/PLE from Kalman filter starts outperforming the AOA-only target localization algorithm, which is corresponding to about 500 iterations under severe noise case.

### 3.4. KF-Based Estimation of TP and PLE for a Moving Target

In the above example, let us consider the target is linearly moving with a velocity *v* (meter per iteration) in ${\left[1,1,1\right]}^{T}$ direction:

(34) $$x_{o}\left[k\right]=x_{o}\left[k-1\right]+v\left[\begin{array}{c} 1\\ 1\\ 1 \end{array}\right],$$

where $x_{o}\left[k\right]$ is target position at the *k*-th iteration. The system matrix in the state-space model, *H*, is replaced with the time-varying model, $H_{k}$, with the updated distance between target and anchors, $d_{i}\left[k\right]=x_{o}\left[k\right]-a_{i}$ in the Equation (26).

**Figure 3:**
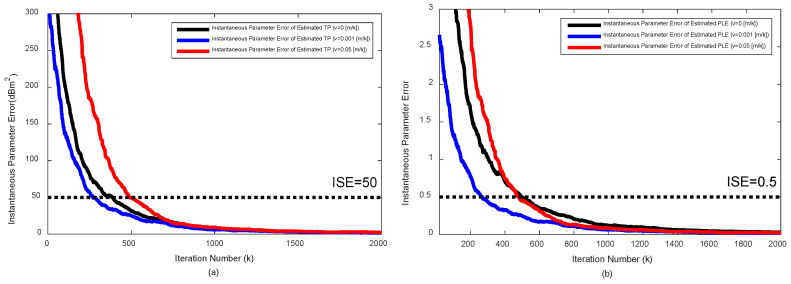
Parameter error vs. iteration for various velocities of the moving target under the severe noise ($\sigma_{n_{i}}=6$ dB, $\sigma_{m_{i}}=\sigma_{v_{i}}=10$ deg): (**a**) for TP, $\hat{\theta }\left(P_{0k}\right)$, and (**b**) for PLE, $\hat{\theta }\left(\gamma_{k}\right)$.

Figure 3 plots the $\hat{\theta }\left({\hat{P}}_{0k}\right)$ and $\hat{\theta }\left({\hat{\gamma }}_{k}\right)$ over the iteration under the severe noise, when the target moves with constant velocities ($v_{static}=0$, $v_{slow}=0.001$, and $v_{fast}=0.05$). Even for the moving target, the reliable TP and PLE estimations that make the hybrid target localization algorithm outperform AOA-only algorithm is possible within 500 iterations.

### 3.5. Refined Estimation of Target Position Using Hybrid Measurements

Once the KF reaches the steady state, the TP and PLE estimates in $z_{k}$ are used to compute the target position by utilizing both RSS and AOA measurements. The EC-WLS algorithm [24] is used to estimate the target position:

(35) $$\begin{array}{c} x_{ECWLS}={\left(A^{T}{\bar{C}}^{-1}A\right)}^{-1}A^{T}{\bar{C}}^{-1}b. \end{array}$$

where $\bar{C}$ is an approximated error covariance matrix, which is given in [24] and

(36) $$\begin{array}{c} A=\left[\begin{array}{c} {\hat{\lambda }}_{1}u_{1}^{T}\\ \vdots \\ {\hat{\lambda }}_{N}u_{N}^{T}\\ c_{1}^{T}\\ \vdots \\ c_{N}^{T}\\ {\left(\cos\left({\hat{\alpha }}_{1}\right)u_{1}-𝟙\right)}^{T}\\ \vdots \\ {\left(\cos\left({\hat{\alpha }}_{N}\right)u_{N}-𝟙\right)}^{T} \end{array}\right],\;b=\left[\begin{array}{c} {\hat{\lambda }}_{1}u_{1}^{T}a_{1}+\hat{\eta }d_{0}\\ \vdots \\ {\hat{\lambda }}_{N}u_{N}^{T}a_{N}+\hat{\eta }d_{0}\\ c_{1}^{T}a_{1}\\ \vdots \\ c_{N}^{T}a_{N}\\ {\left(\cos\left({\hat{\alpha }}_{1}\right)u_{1}-𝟙\right)}^{T}a_{1}\\ \vdots \\ {\left(\cos\left({\hat{\alpha }}_{N}\right)u_{N}-𝟙\right)}^{T}a_{N} \end{array}\right],{\hat{\lambda }}_{i}:=10^{\frac{{\hat{P}}_{i}}{10\hat{\gamma }}},\hat{\eta }:=10^{\frac{{\hat{P}}_{0}}{10\hat{\gamma }}} \end{array}$$

In summary, the proposed hybrid target localization algorithm, summarized in Algorithm 1, does not depend on the prior information on the unknown parameters, i.e., TP and PLE.
**Algorithm 1** Proposed hybrid target localization algorithm under unknown TP and PLE.**1.** Estimate the target position from AOA measurements. **Solve**
${\hat{x}}_{AOA}={\left(B^{T}C_{A}^{-1}B\right)}^{-1}B^{T}C_{A}^{-1}c$ (24) **2.** Estimate the TP and PLE using the KF. **i.** Initialize $z_{0}=z_{LS}$ and $Q_{0}=I_{2}$. **ii.** Predict and update $z_{k}$, $K_{k}$ and $Q_{k}$. (30) **for**
k=1,⋯,K Predict: $z_{k|k-1}=z_{k-1}$ $Q_{k|k-1}=Q_{k-1}$ Update: $z_{k}=z_{k|k-1}+K_{k}\left(p_{k}-Hz_{k|k-1}\right)$ $K_{k}=Q_{k|k-1}H^{T}{\left(HQ_{k|k-1}H^{T}+R\right)}^{-1}$ $Q_{k}=\left(I-K_{k}H\right)Q_{k|k-1}$ **end** **3.** Estimate the target position using EC-WLS with the estimated parameters $z_{K}$ (TP and PLE). **Solve**
${\hat{x}}_{ECWLS}={\left(A^{T}{\bar{C}}^{-1}A\right)}^{-1}A^{T}{\bar{C}}^{-1}b$ (35)

## 4. Complexity Comparison

In this section, we compare the computational complexities of existing mentioned target localization algorithms with unknown TP and PLE. The proposed method has three parts for target localization; estimation of initial target, estimation of TP and PLE, and estimation of final target. The complexity for initial target estimation using only AOA measurements is O(N). When the Kalman filter is applied to TP and PLE, each step the complexity is $O(N^{3})$ due to the matrix inversion in $K_{k}$. Finally, the complexity for final target estimation is again O(N). Let *K* be the total number of iterations. The complexity of the proposed method for target localization is $KO(N^{3})$.

Table 1 presents the complexities of existing hybrid target localization algorithms with unknown TP and PLE. In the table *M* denotes the mesh size (M≫N) used in evaluating the cost function of WLLS, and $K_{max}$ is the maximum number of steps in the bisection procedure used in SR-WLS. The proposed method has the second highest complexity. However, the proposed algorithm is the only algorithm that can deal with both unknown TP and unknown PLE. Furthermore, the $O(N^{3})$ computational burden in each iteration is acceptable, considering the computational power of modern DSPs.

## 5. Performance Results

This section details the performance of our proposed algorithm in numerical simulations under experimental settings utilized in most of previous works. Target and anchor nodes are arbitrarily placed inside a box with an edge length B=15 m for each Monte Carlo run. $d_{0}$ is set to 1 m; the true TP, $P_{0}$, is set to −10 dBm; the true PLE, γ, is set to 2.5 for all anchors as a fixed value; the number of Monte Carlo simulations, $M_{c}$, is set to 50,000. The maximum iteration number of the Kalman filter, *K*, is set to 1000. The performance is measured by the root-mean-square error (RMSE), which is defined as

(37) $$RMSE=\sqrt{\frac{1}{M_{c}}\sum_{i=1}^{M_{c}}{\left\|x_{oi}-{\hat{x}}_{i}\right\|}^{2}},$$

where ${\hat{x}}_{i}$ is the target location estimate, $x_{oi}$, at the *i*-th run.

We compare the RMSEs of the proposed algorithm with those of the AOA-only algorithm based on EC-WLS (24), SR-WLS with unknown TP reported in [32], and TT-WLS (with unknown TP) reported in [33]. As a reference, the performance of the EC-WLS algorithm with known TP and PLE in [24] is also compared with that of the proposed algorithm.

**Figure 4:**
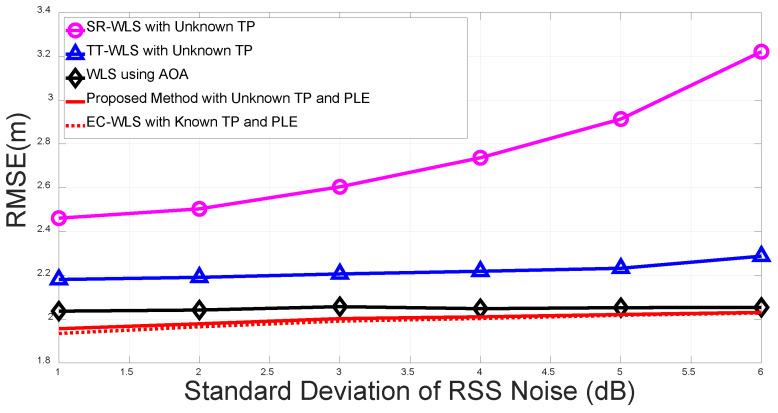
Root mean square error (RMSE) vs. standard deviation for the RSS noise.

Figure 4 presents a plot of the RMSEs of the proposed method and the existing methods as standard deviations of the RSS noise, $\sigma_{n_{i}}^{2}$, which increases under $\sigma_{m_{i}}=\sigma_{v_{i}}=10$ deg for a fixed number of anchor nodes, N=4. The simulation results indicate that AOA-only EC-WLS outperforms the conventional method with unknown TP, whereas our proposed method with unknown TP and PLE outperforms the AOA-only method as well as the conventional hybrid target position algorithms with unknown TP; the performance of the proposed method is bounded by the performance of EC-WLS with known TP and PLE. The difference in the performances of the proposed method and the EC-WLS with known TP and PLE was found to be negligible.

**Figure 5:**
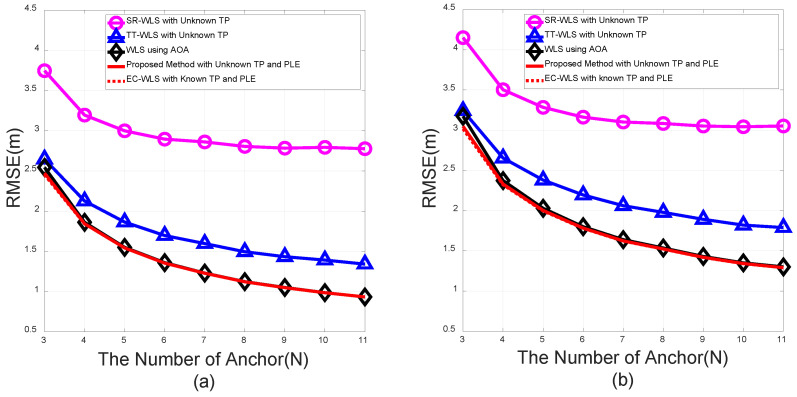
RMSE vs. the number of nodes under noise variance dispersion with (**a**) mild differences and (**b**) large differences.

In practice, measurement noises typically feature inhomogeneous noise variance among the anchor nodes. Figure 5a presents a case with a small deviation in the standard deviation of the noise as the number of anchor nodes, *N*, increases; the measurement noises are modeled as uniform distributions over the nodes as $\sigma_{n_{i}}$∼Unif[3,9] (dB), $\sigma_{m_{i}}$∼Unif[6,12], and $\sigma_{v_{i}}$∼Unif[6,12] deg. Figure 5b depicts a case with a large deviation in the measurement noise variance as the number of anchor nodes, *N*, increases; $\sigma_{n_{i}}=\mathrm{Unif}\left[1,10\right]$ (dB), $\sigma_{m_{i}}$∼Unif[1,20], and $\sigma_{v_{i}}$∼Unif[1,20] deg. In both cases, the proposed algorithm with unknown TP and PLE outperforms the AOA-only target localization method as well as the conventional hybrid methods with unknown TP.

**Figure 6:**
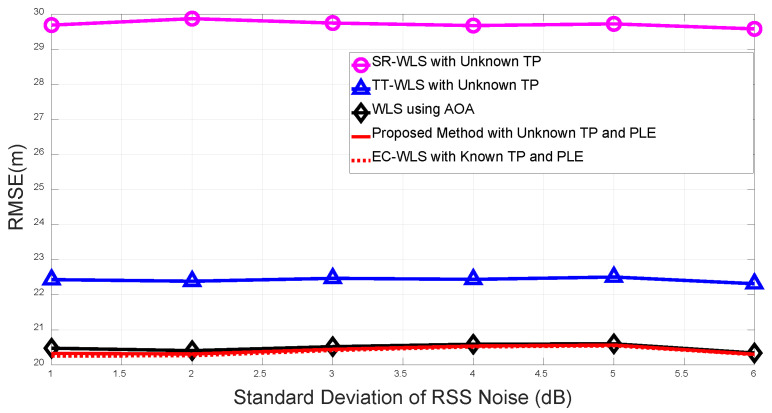
RMSE vs. standard deviation of received signal strength (RSS) noise on a large scale.

Finally, we compare the performance of the proposed algorithm on a large scale. Figure 6 shows the RMSEs of the proposed method, conventional hybrid methods, and the AOA-only method for an increase in the RSS noise, with the edge length B=150 m and a fixed number of anchor nodes N=4. The noise level is set to $\sigma_{m_{i}}=\sigma_{v_{i}}=10$ deg. The results reveal that the proposed method performs better than all the conventional hybrid methods as well as the AOA-only method on a large scale.

## 6. Practical Considerations and Limitations

Since most hybrid localization algorithms estimate the target position at the central node (processor) using the gathered RSS/AOA data from all nodes in the sensor network via a communication link such as IEEE 802.11 (refer related references in [23]), the proposed algorithm can be applied for existing hybrid target localization algorithms by simple software update in the center node. For the situations where the transmit power (TP) and path-loss exponent (PLE) are time-varying due to battery consumption or meteorological changes, the proposed algorithm can update exact TP and PLE information in parallel with the conventional hybrid target localization algorithm and thus improve overall performance of the hybrid localization algorithm. When the TP and PLE are completely unknown, for example, a military surveillance system operating with low energy consumption restriction in hostile environment and, therefore, only AOA measurements are used for target estimation, the proposed one can estimate TP and PLE and, consequently, improves the target accuracy by utilizing RSS measurements.

However, the proposed algorithm relies on relatively reliable initial estimation of the target position. When the initial estimation is corrupted by severe angle noise due to possible multipath propagation (non-line-of-sight) or multiple inference, the proposed algorithm may not recover from the bad initialization of the TP and PLE. However, in such cases, all existing hybrid algorithms are subject to fail as well. Furthermore, possible TP/PLE acquisition delay due to the convergence time of Kalman filter in the presence of poor initialization can be problematic for a certain application requiring rapid target estimation.

Nonetheless, the proposed algorithm enhances the accuracy of the estimated target. The proposed algorithm produces almost the same performance with the state-of-the-art algorithm with known TP and PLE [24], while conventional hybrid algorithm relying on known TP and PLE cannot be applied. Furthermore, the proposed algorithm outperforms other existing TP and PLE estimation algorithms [30,31,32,33] as confirmed in Section 5.

In order to verify the robust performance of the proposed algorithm in realistic adversary situations, we have considered a multipath propagation scenario and a multiple interference environment.

**Figure 7:**
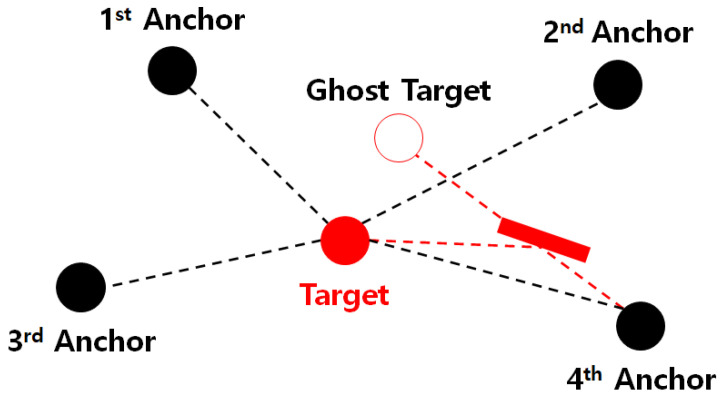
Distortion model of multipath propagation in wireless sensor networks (WSNs).

**Figure 8:**
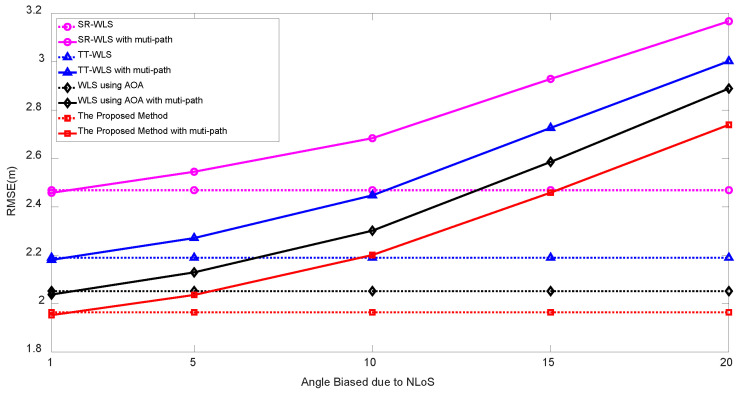
RMSE vs. angle bias due to multipath propagation at the 4th anchor.

Figure 7 illuminates an example of multipath propagation. Since the 4-th anchor received the signal traveling through a non-line-of-sight as well, the AOA/RSS measurements of the 4-th anchor are subject to be biased by the ghost target. Figure 8 compares the performance of TP/PLE estimating target localization algorithms in the presence of multipath. The measurements noises are set to $\sigma_{n}=1$ dB, $\sigma_{m}=\sigma_{v}=10^{\circ }$ and we have assumed that the 4-th anchor suffers from multipath distortion. The RSS measurement of the 4-th anchor has 1 dB loss and the angle measurements are biased from 1${}^{\circ }$ to $20^{\circ }$ due to multipath propagation. Figure 8 clearly shows that the resulting RMSE performances are degraded in comparison with the absence of multipath (dotted line). However, the proposed algorithm still yields the best performance among existing TP/PLE estimating hybrid localization algorithms.

We also consider the multiple interference. The multiple interference are caused by additive noises from other communication networks and statistically modeled as inverse Gaussian distribution as studied in [37,38], where the inverse Gaussian pdf with mean μ and the shape parameter ρ is given as

(38) $$\begin{array}{c} f\left(x;\mu ,\rho \right)=\sqrt{\frac{\rho }{2\pi x^{3}}}\exp\left(-\frac{\rho {\left(x-\mu \right)}^{2}}{2\mu^{2}x}\right) \end{array}$$

Since the conventional methods and the proposed algorithm are developed under the zero mean Gaussian noise assumption, non-Gaussian interference with nonzero mean would degrade the performance.

**Figure 9:**
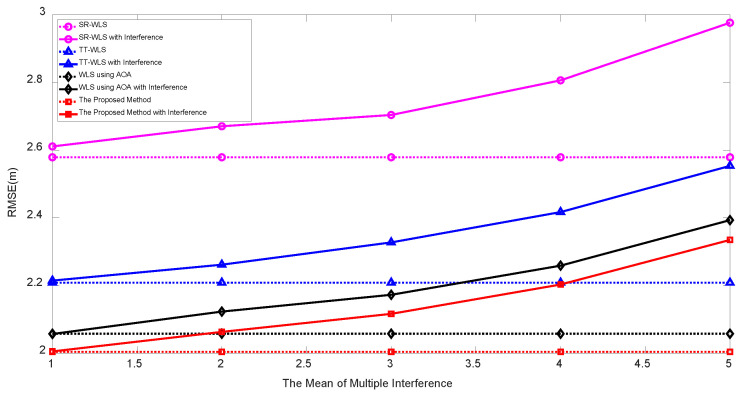
RMSE vs. the mean of inverse-Gaussian interference for fixed ρ=25.

Figure 9 shows the simulation results under the basic measurement noise setting $\sigma_{n}=3$ dB and $\sigma_{m}=\sigma_{v}=10^{\circ }$ with inverse Gaussian interference noise with the mean varying from 1 to 5 dB with fixed shape parameter ρ=25 (from 0.4 to 5 in terms of variance). As the mean of interference increases the RMSE performance of all algorithms are degraded in comparison with absence of interference (dotted line). However the proposed algorithm yields the best performance among existing TP/PLE estimating hybrid localization algorithms.

## 7. Conclusions

Herein, we presented a novel hybrid localization algorithm under unknown TP and PLE. The initial target position was estimated via the AOA-only EC-WLS method, and the TP and PLE were estimated by using a KF. After the TP and PLE are estimated, the EC-WLS algorithm is used to incorporate the RSS measurements. The proposed algorithm outperforms the conventional hybrid target localization methods for unknown TP as well as the AOA-only EC-WLS method. Furthermore, we prove that the proposed method almost achieves a performance comparable to that of the EC-WLS algorithm with known TP and PLE. The proposed algorithm can be further extended for the case where each node has a different PLE value or multiple targets have different TP values, which is left for future research. 

## Figures and Tables

**Table 1:** Complexities of target location algorithms with unknown transmit power (TP) and PLE.

Algorithm	Description	Complexity
SOCP	The SOCP method in case for unknown TP in [30]	$KO(N^{3.5})$
WLLS	The WLLS method in case for unknown PLE in [31]	KO(MN)
SR-WLS	The SR-WLS method in case for unknown TP in [32]	$2O(K_{max}N)$
TP-WLS	The TP-WLS method in case for unknown TP in [33]	O(N)
KF-ECWLS	The proposed method in case for unknown TP and PLE	$KO(N^{3})$

## Appendix A. Approximated Error Covariance Matrix for AOA Measurements

The approximated error covariance matrix for AOA measurements is expressed as

(A1) $$\begin{array}{c} \begin{array}{cc} C_{A}= & \left[\begin{array}{cc} \mathrm{Var}({\hat{\varepsilon }}_{i2}) & \mathrm{Cov}({\hat{\varepsilon }}_{i2},{\hat{\varepsilon }}_{i3})\\ \mathrm{Cov}({\hat{\varepsilon }}_{i2},{\hat{\varepsilon }}_{i3}) & \mathrm{Var}({\hat{\varepsilon }}_{i3}) \end{array}\right] \end{array} \end{array}$$

where, from the computation of covariances in [24],

(A2) $$\begin{array}{cc} \mathrm{Var}({\hat{\varepsilon }}_{i2}) & ={\hat{r}}_{i}^{2}{\hat{\sigma }}_{m_{i}}^{2} \end{array}$$

(A3) $$\begin{array}{c} \begin{array}{cc} \mathrm{Var}({\hat{\varepsilon }}_{i3}) & ={\left(\cos\left(2{\tilde{\alpha }}_{i}\right){\hat{r}}_{i}-\sin\left(2{\tilde{\alpha }}_{i}\right){\hat{d}}_{iz}\right)}^{2}{\hat{\sigma }}_{v_{i}}^{2}+\frac{1}{8}{\left(\sin\left(2{\tilde{\alpha }}_{i}\right){\hat{r}}_{i}\right)}^{2}{\hat{\sigma }}_{m_{i}}^{4}\\ & \;+2{\left(\sin\left(2{\tilde{\alpha }}_{i}\right){\hat{r}}_{i}-\cos\left(2{\tilde{\alpha }}_{i}\right){\hat{d}}_{iz}\right)}^{2}{\hat{\sigma }}_{v_{i}}^{4} \end{array} \end{array}$$

(A4) $$\begin{array}{c} \begin{array}{cc} \mathrm{Cov}({\hat{\varepsilon }}_{i2},{\hat{\varepsilon }}_{i3}) & =0 \end{array} \end{array}$$

The components in $C_{A}$ are obtained using the initial LS target position solution (without weighted),

(A5) $$\begin{array}{c} x_{LS\_AOA}={\left(B^{T}B\right)}^{-1}B^{T}c, \end{array}$$

(A6) $$\begin{array}{c} \begin{array}{cc} {\tilde{\phi }}_{i} & =\tan^{-1}\left(\frac{x_{LS\_AOA_{y}}-a_{iy}}{x_{LS\_AOA_{x}}-a_{ix}}\right),\\ {\tilde{\alpha }}_{i} & =\cos^{-1}\left(\frac{x_{LS\_AOA_{z}}-a_{iz}}{\left\|x_{LS\_AOA}-a_{i}\right\|}\right), \end{array}\;\mathrm{for}\;i=1,\cdots ,N. \end{array}$$

(A7) $$\begin{array}{c} \begin{array}{cc} {\hat{d}}_{iz} & =x_{LS\_AOA_{z}}-a_{iz},\\ {\hat{r}}_{i} & =\sqrt{{\left(x_{LS\_AOA_{x}}-a_{ix}\right)}^{2}+{\left(x_{LS\_AOA_{y}}-a_{iy}\right)}^{2}} \end{array} \end{array}$$

(A8) $$\begin{array}{c} \begin{array}{cc} {\hat{\sigma }}_{m_{i}}^{2} & =\frac{1}{N}\sum_{i=1}^{N}{\left({\hat{\phi }}_{i}-\tan^{-1}\left(\frac{x_{LS\_AOA_{y}}-a_{iy}}{x_{LS\_AOA_{x}}-a_{ix}}\right)\right)}^{2}\\ {\hat{\sigma }}_{v_{i}}^{2} & =\frac{1}{N}\sum_{i=1}^{N}{\left({\hat{\alpha }}_{i}-\cos^{-1}\left(\frac{x_{LS\_AOA_{z}}-a_{iz}}{\left\|x_{LS\_AOA}-a_{i}\right\|}\right)\right)}^{2} \end{array} \end{array}$$

## References

[1] Salman N., Ghogho M., Kemp A.H. (2014). Optimized low complexity sensor node positioning in wireless sensor networks. IEEE Sens. J..

[2] Dai W., Shen Y., Win M.Z. (2011). Energy-efficient network navigation algorithms. IEEE J. Sel. Areas Commun..

[3] Bartoletti S., Dai W., Conti A., Win M.Z. (2015). A mathematical model for wideband ranging. IEEE J. Sel. Top. Signal Process..

[4] Masazade E., Niu R., Varshney P.K., Keskinoz M. (2010). Energy aware iterative source localization for wireless sensor networks. IEEE Trans. Signal Process..

[5] Tomic S., Beko M., Dinis R., Tuba M., Bacanin N. (2017). RSS-AoA-Based Target Localization and Tracking in Wireless Sensor Networks.

[6] Shen Y., Mazuelas S., Win M.Z. (2012). Network navigation: Theory and interpretation. IEEE J. Sel. Areas Commun..

[7] Tomic S., Beko M., Dinis R. (2015). RSS-based localization in wireless sensor networks using convex relaxation: Noncooperative and cooperative schemes. IEEE Trans. Veh. Technol..

[8] Patwari N., Ash J., Kyperountas S., Hero A.O., Moses R.L., Correal N.S. (2005). Locating the nodes: Cooperative localization in wireless sensor networks. IEEE Signal Process. Mag..

[9] Guvenc I., Chong C.C. (2009). A survey on TOA based wireless localization and NLOS mitigation techniques. IEEE Commun. Surv. Tutor..

[10] Win M.Z., Conti A., Mazuelas S., Shen Y., Gifford W.M., Dardari D., Chiani M. (2010). Network localization and navigation via cooperation. IEEE Commun. Mag..

[11] Patwari N. (2005). Location Estimation in Sensor Networks. Ph.D. Thesis.

[12] Li X. (2007). Collaborative localization with received-signal strength in wireless sensor networks. IEEE Trans. Veh. Technol..

[13] Ouyang R.W., Wong A.K.S., Lea C.T. (2010). Received signal strength-based wireless localization via semidefinite programming: Noncooperative and cooperative. IEEE Trans. Veh. Technol..

[14] Wang G., Yang K. (2011). A new approach to sensor node localization using RSS measurements in wireless sensor networks. IEEE Trans. Wirel. Commun..

[15] Li M., Lu Y. Angle-of-Arrival Estimation for Localization and Communication in Wireless Networks. Proceedings of the 16th European Signal Processing Conference (EUSIPCO 2008).

[16] Shao H.J., Zhang X.P., Wang Z. (2014). Efficient closed-form algorithms for AOA based self-localization of sensor nodes using auxiliary variables. IEEE Signal Process..

[17] Wang Y., Ho K.C. (2015). An asymptotically efficient estimator in closed-form for 3-D AOA localization using a sensor network. IEEE Trans. Wirel. Commun..

[18] Yu K. (2007). 3-D localization error analysis in wireless networks. IEEE Trans. Wirel. Commun..

[19] Gazzah L., Najjar L., Besbes H. Selective Hybrid RSS/AOA Weighting Algorithm for NLOS Intra Cell Localization. Proceedings of the 2014 IEEE Wireless Communications and Networking Conference (WCNC).

[20] Chan Y.T., Chan F., Read W., Jackson B.R., Lee B.H. Hybrid Localization of an Emitter by Combining Angle-of-Arrival and Received Signal Strength Measurements. Proceedings of the 2014 IEEE 27th Canadian Conference on Electrical and Computer Engineering (CCECE).

[21] Biswas P., Aghajan H., Ye Y. Semidefinite Programming Algorithms for Sensor Network Localization Using Angle of Arrival Information. Proceedings of the Asilomar Conference on Signals, Systems, and Computers.

[22] Tomic S., Marikj M., Beko M., Dinis R., Órfão N. Hybrid RSS-AoA Technique for 3-D Node Localization in Wireless Sensor Networks. Proceedings of the 11th International Wireless Communications and Mobile Computing Conference (IWCMC 2015).

[23] Tomic S., Beko M., Dinis R., Bernardo L. (2018). On target localization using combined RSS and AoA measurements. Sensors.

[24] Kang S.Y., Kim T.H., Chung W.Z. (2020). Hybrid RSS/AOA localization using approximated weighted least square in wireless sensor networks. Sensors.

[25] Vaghefi R., Gholami M., Buehrer R., Ström E. (2013). Cooperative received signal strength-based sensor localization with unknown transmit powers. IEEE. Trans. Signal Proc..

[26] Huang J., Liu P., Lin W., Gui G. (2016). RSS-based method for sensor localization with unknown transmit power and uncertainty in path loss exponent. Sensors.

[27] Wang G., Chen H., Li Y., Jin M. (2012). On received-signal-strength based localization with unknown transmit power and path loss exponent. IEEE. Wirel. Comm. Lett..

[28] Tomic S., Beko M., Dinis R., Lipovac V., Dimimc G. RSS-based Localization in Wireless Sensor Networks with Unknown Transmit Power and Path Loss Exponent using SDP Relaxation. Proceedings of the WSEAS International Conference on Applied Electromagnetics, Wireless and Optical Communications (ELEC-TROSCIENCE).

[29] Lin L., So H.C., Chan Y.T. (2014). Received signal strength based positioning for multiple nodes in wireless sensor networks. Digit. Signal Proc..

[30] Tomic S., Beko M., Dinis R. (2016). Distributed RSS-AoA based localization with unknown transmit powers. IEEE Wirel. Commun. Lett..

[31] Khan M.W., Salman N., Kemp A.H., Mihaylova L. (2016). Localisation of sensor nodes with hybrid measurements in wireless sensor networks. Sensors.

[32] Tomic S., Beko M., Dinis R. (2017). 3-D target localization in wireless sensor network using RSS and AoA measurement. IEEE Trans. Veh. Technol..

[33] Tomic S., Beko M., Dinis R., Montezuma P. (2016). A closed-form solution for RSS/AoA target localization by spherical coordinates conversion. IEEE Wirel. Commun. Lett..

[34] Park S.B., Gil M.S., Im H.S. (2019). Measurement Noise Recommendation for Efficient Kalman Filtering over a Large Amount of Sensor Data. Sensors.

[35] Kay S.M. (1993). Fundamentals of Statistical Signal Processing: Estimation Theory.

[36] Rappaport T.S. (1996). Wireless Communications: Principles and Practice.

[37] Kountouris M., Pappas N. Approximating the Interference Distribution in Large Wireless Networks. Proceedings of the 2014 11th International Symposium on Wireless Communications Systems (ISWCS).

[38] Noreen U., Bounceur A., Clavier L. Modeling Interference for Wireless Sensor Network Simulators. Proceedings of the International Conference on Future Networks and Distributed Systems.

